# Medical Convention for Revising the Pharmacopœia of the United States

**Published:** 1859-05

**Authors:** Geo. B. Wood

**Affiliations:** President of the Convention of 1850; Philadelphia


### MEDICAL CONVENTION FOR REVISING THE PHARMACOPÆIA OF THE UNITED STATES.

The Medical Convention for revising the Pharmacopœia, which met at Washington in May, 1850, provided for assembling a Convention for the purpose, in the year 1860, by the following resolutions:

"1. The President of the Convention shall, on the first day of May, 1859, issue a notice, requesting the several incorporated State Medical Societies, the incorporated State Medical Colleges, the incorporated Colleges of Physicians and Surgeons, and the incorporated Colleges of Pharmacy, throughout the United States, to elect a number of delegates, not exceeding three, to attend a general Convention, to be held at Washington on the first Wednesday in May, 1860.

"2. The several incorporated bodies, thus addressed, shall also be requested by the President to submit the Pharmacopœia to a careful revision, and to transmit the result of their labors, through their delegates, or through any other channel, to the next Convention.

"3. The several medical and pharmaceutical bodies shall be further requested to transmit to the President of the Convention, the names and residences of their respective delegates, as soon as they shall have been appointed, a list of whom shall be published, under his authority, for the information of the medical public, in the newspapers and medical journals, in the month of March, 1860."

In accordance with the above resolutions, the undersigned hereby requests the several bodies mentioned to appoint delegates, not exceeding three in number, to represent them in a Convention, for revising the Pharmacopœia of the United States, to meet at Washington on the first Wednesday in May, 1860; and would also call the attention of these bodies to the second and third resolutions, and request compliance with the suggestions therein contained.

GEO. B. WOOD,
*President of the Convention of* 1850.

PHILADELPHIA, May 1st, 1859.

